# Fundamental Motor Skills and Motor Competence in Children and Adolescents with Autism Spectrum Disorder (ASD): A Narrative Review

**DOI:** 10.3390/children13040520

**Published:** 2026-04-08

**Authors:** Katerina Asonitou, Melina Kottara, Sophia Charitou, Dimitra Koutsouki

**Affiliations:** School of Physical Education & Sport Science, National and Kapodistrian University of Athens, 17237 Athens, Greece; mekottar@phyed.duth.gr (M.K.); sofhar@phed.uoa.gr (S.C.); dkoutsou@phed.uoa.gr (D.K.)

**Keywords:** gross motor skills, fine motor skills, motor competence, perceived motor competence, physical activity, adapted physical education

## Abstract

**Background/Objectives:** Children and adolescents on the autism spectrum often experience delays in both gross and fine motor skills, which can limit their participation in physical activity and everyday tasks. **Methods:** This narrative review synthesizes evidence from 88 peer-reviewed studies examining fundamental motor skills, broader motor competence, and perceived motor competence in individuals aged 3–18 years with a formal diagnosis of autism. **Results:** Across the literature, children with autism consistently demonstrate lower proficiency in locomotor and object control skills compared with their typically developing peers, while perceived competence emerges as an important factor influencing motivation and engagement. Intervention studies—most commonly school-based or structured physical activity programs—generally report short-term improvements in motor performance, although outcomes vary depending on study design, dosage, and assessment tools. The review also highlights substantial methodological heterogeneity and a notable lack of evidence concerning adolescents, underscoring the need for longitudinal and developmentally sensitive research. **Conclusions:** Practical implications are discussed for creating supportive movement environments in educational and adapted physical activity settings. This review follows a narrative synthesis approach informed by a structured search strategy.

## 1. Introduction

Motor development is a fundamental dimension of children’s growth, shaping how they explore their environment, interact with others, and participate in both spontaneous play and structured physical activity. Fundamental motor skills (FMSs)—locomotor, object control, and stability skills—do not simply unfold on their own; although maturation provides the biological foundation, the refinement of these skills depends heavily on meaningful practice, repetition, and supportive learning environments that allow children to experiment, fail, and gradually master movement patterns [1]. Building on this foundation, motor competence (MC) reflects the quality, coordination, and adaptability of movement, emerging from the continuous interplay between biological predispositions and the opportunities afforded by the environment [2]. Perceived motor competence (PMC) adds a motivational layer to this developmental picture, influencing children’s confidence, their willingness to participate, and ultimately their long-term engagement in physical activity [3,4].

Autism spectrum disorder (ASD) is a neurodevelopmental condition characterized by differences in social communication, restricted interests, and repetitive patterns of behavior [5]. Motor difficulties are increasingly recognized as a common feature of ASD, with many individuals presenting hypotonia, dyspraxia, and coordination challenges that affect both gross and fine motor development [6]. Co-occurring conditions such as attention-deficit/hyperactivity disorder (ADHD), developmental coordination disorder (DCD) traits, and sensory processing differences are also prevalent and may further influence motor competence and participation in physical activity [7]. These characteristics are particularly relevant to the present review, which examines fundamental motor skills, broader motor competence, and perceived motor competence in children and adolescents on the autism spectrum.

Within autism spectrum disorder (ASD), motor difficulties are increasingly recognized not as peripheral challenges but as core developmental features that often appear early and persist across childhood [8,9]. A substantial body of research shows that children on the spectrum typically demonstrate lower proficiency in both gross and fine motor skills, reduced coordination, and overall lower levels of motor competence compared with their typically developing peers [10,11]. These motor challenges can have cascading effects: they may limit participation in physical activity, restrict opportunities for social interaction, and contribute to broader developmental disparities in areas such as self-regulation, academic engagement, and adaptive functioning [12,13].

In recent years, research has begun to look beyond observable motor behaviors to examine the cognitive mechanisms that underpin motor performance in ASD. Neuropsychological evidence highlights the role of visuospatial working memory, attentional control, planning, and decision-making in shaping fundamental movement skills [14]. Perspectives grounded in the Planning, Attention, Simultaneous, and Successive (PASS) cognitive processing theory further emphasize that motor tasks place cognitive demands on learners, suggesting that assessment and intervention approaches must consider both movement and cognition as intertwined processes [15]. At the same time, studies on physical activity interventions point to the multidimensional benefits of movement-based programs, which can enhance not only motor proficiency but also executive functioning, social skills, and overall quality of life for children and adolescents with neurodevelopmental conditions [16,17].

Despite these advances, the literature remains fragmented. Studies differ widely in their assessment tools, sample characteristics, and intervention designs, making it difficult to draw unified conclusions or identify consistent developmental patterns. Existing reviews often focus on isolated aspects of motor development—such as gross motor performance or intervention effectiveness—without integrating FMSs, MC, and PMC into a coherent developmental framework [18,19]. Adolescents, in particular, remain underrepresented in the literature, and few reviews explicitly address how motor-related findings can inform inclusive physical education practice or adapted physical activity programming [19].

Against this backdrop, the present narrative review seeks to synthesize evidence on FMSs, MC, and PMC in children and adolescents with ASD, offering a more integrated understanding of motor development across this population and highlighting implications for educational and therapeutic practice. To address the fragmentation observed in the previous literature, the present review adopts an integrative conceptual model linking fundamental motor skills, motor competence, perceived motor competence, cognitive processes, and environmental opportunities. This model positions motor development as a dynamic system shaped by interactions among movement proficiency, motivation, cognition, and context [14,15].

Despite the growing body of research on motor development in ASD, the literature remains conceptually fragmented. Studies examining fundamental motor skills, broader motor competence, and perceived motor competence often operate in parallel rather than in dialogue, resulting in a piecemeal understanding of how these domains interact across development. Existing reviews tend to focus on isolated constructs or specific intervention outcomes, leaving limited insight into how motor performance, motivation, and cognitive processes may influence one another over time. This lack of integration makes it difficult to identify developmental patterns, interpret inconsistencies across studies, or translate findings into coherent educational and therapeutic practices.

To address this fragmentation, the present review adopts an integrative developmental framework that links FMSs, MC, and PMC within a broader system shaped by cognitive processes and environmental opportunities. In this model, fundamental motor skills form the foundation for more complex movement patterns [1,2]; motor competence reflects the quality and adaptability of these skills [1,2]; and perceived motor competence influences children’s motivation, persistence, and engagement in physical activity [3,4]. Cognitive factors such as attention, planning, and visuospatial processing interact with these domains [14,15], while environmental supports—such as structured instruction, opportunities for practice, and inclusive physical education settings—shape the conditions under which motor development unfolds [1,2,16]. By making these relationships explicit, the framework provides a structure for interpreting the diverse findings reported in the literature.

Although this review draws on a structured search strategy to ensure transparency and breadth, it is not intended to function as a systematic or scoping review. The considerable heterogeneity across study designs, assessment tools, age groups, and intervention approaches in the ASD motor literature makes a rigid systematic methodology less suitable for the purposes of this work. Instead, the review adopts a conceptually driven narrative approach, which allows for a more flexible and integrative synthesis of findings across fundamental motor skills, motor competence, and perceived motor competence. This epistemological stance aligns with the primary aim of the manuscript: to bring together diverse strands of evidence and interpret them within a coherent developmental framework, rather than to evaluate intervention effectiveness or produce an exhaustive methodological mapping of the field.

### Aim of the Study

The purpose of this narrative review is to bring together the diverse body of research examining fundamental motor skills (FMSs), motor competence (MC), and perceived motor competence (PMC) in children and adolescents with autism spectrum disorder (ASD). Although motor difficulties are well documented and tend to persist across development, much of the existing literature treats these constructs separately, resulting in a fragmented picture of motor development in ASD. Recent reviews confirm this fragmentation, noting that FMSs, MC, and PMC are rarely examined together and often assessed in isolation [10,17,18].

Recent work has drawn attention to the cognitive processes that underpin motor performance—such as visuospatial working memory, attention, planning, and decision-making—and how these mechanisms may shape the motor profiles of children on the spectrum [14]. At the same time, evidence from physical activity and sport-based interventions highlights the broader developmental benefits of movement, including gains in executive functioning, social behavior, and overall quality of life [16,17].

Against this backdrop, the present review aims to:

- examine how FMSs, MC, and PMC have been assessed in children and adolescents with ASD;
- compare motor performance between individuals with ASD and their typically developing peers;
- synthesize findings from intervention studies targeting motor development;
- consider developmental differences across childhood and adolescence;
- and outline practical implications for inclusive physical education and adapted physical activity settings.

We clarify that the primary purpose of the review is conceptual integration supported by descriptive synthesis. The aim is not to conduct a formal evaluation of intervention effectiveness, but to bring together diverse strands of evidence on FMSs, MC, and PMC within a unified developmental framework [10,17,18].

By integrating these strands of evidence, the review seeks to clarify conceptual relationships, identify methodological gaps, and support the design of interventions that more effectively address the motor and motivational needs of children and adolescents on the autism spectrum.

## 2. Methods

This review follows a narrative synthesis approach guided by a structured search strategy. The decision to adopt a narrative rather than a systematic or scoping methodology reflects the substantial variability observed in the ASD motor literature, including differences in study aims, measurement tools, sample characteristics, and intervention formats. Such heterogeneity limits the feasibility and usefulness of applying rigid systematic procedures or formal quality appraisal tools. Instead, the narrative approach allows for a more conceptually oriented integration of findings, with emphasis on developmental relationships among FMSs, MC, and PMC. The structured search enhances transparency and comprehensiveness, but it is not intended to meet the procedural requirements of a systematic review.

### 2.1. Search Strategy

A structured literature search was conducted between March 2020 and October 2025 across four major electronic databases: PubMed, ScienceDirect, ProQuest, and Google Scholar. The search focused on peer-reviewed studies published in English that examined fundamental motor skills (FMSs), motor competence (MC), or perceived motor competence (PMC) in children and adolescents diagnosed with autism spectrum disorder (ASD). The decision to prioritize recent literature was intentional, as contemporary studies reflect updated diagnostic criteria, modern assessment tools, and current intervention approaches that differ substantially from earlier work. This timeframe reflects the shift toward updated diagnostic criteria, contemporary assessment tools, and modern intervention methodologies. Earlier foundational studies were included only when they provided essential theoretical or developmental context [16,18].

Search terms were combined using Boolean operators and adapted for each database to ensure sensitivity and relevance. Keywords included: “fundamental motor skills,” “motor competence,” “gross motor skills,” “fine motor skills,” “perceived motor competence,” “physical activity,” “autism spectrum disorder,” “ASD,” “intervention,” “children,” and “adolescents.” Two additional articles published in 2024 and 2025 were identified through manual reference checking and were included because they met all eligibility criteria.

Although the systematic search focused on studies published between 2020 and 2025, several influential earlier works were also included because they provide essential theoretical and developmental context for understanding FMSs, MC, and PMC in children and adolescents with ASD.

To enhance methodological transparency, the full search strings for all databases are presented in Table 1. Providing the exact Boolean combinations allows readers to understand how the search was conducted and ensures reproducibility, while keeping the main text focused and readable.

In addition to the structured search conducted between 2020 and 2025, earlier influential studies were also included when they provided essential theoretical, developmental, or methodological context for understanding FMSs, MC, and PMC in ASD. Because this review follows a narrative synthesis approach, the final body of evidence reflects both the results of the structured search and key foundational studies published before 2020. Accordingly, Table 2 and Table 3 present all studies that directly examined FMSs, MC, or PMC, regardless of publication year, to ensure conceptual completeness and thematic coherence.

### 2.2. Study Selection

All records retrieved from the database search were imported into a reference management system, where duplicates were removed. Titles and abstracts were then screened to identify studies relevant to FMSs, MC, or PMC in children with ASD. Initial screening was conducted by one reviewer, with all uncertain cases discussed and resolved in consultation with a second reviewer. The selection process unfolded in three stages.

Stage 1: Title and Abstract Screening

In the first stage, titles and abstracts were screened for relevance based on predefined eligibility criteria. Articles clearly unrelated to motor development, ASD, or the target age range were excluded.

Stage 2: Full-Text Review

In the second stage, full-text articles were reviewed and categorized as “include,” “exclude,” or “uncertain.” Although screening was primarily conducted by one reviewer, studies marked as “uncertain” were discussed with a second reviewer until consensus was reached. This dual-reviewer approach, while modest, is considered acceptable in narrative reviews where methodological heterogeneity is expected.

Stage 3: Reference List Screening

In the third stage, reference lists of included studies were manually searched to identify additional eligible articles. Studies known to the authors were also evaluated using the same criteria to ensure transparency and avoid selection bias.

Although the initial screening of titles and abstracts was conducted by one reviewer, all studies categorized as “uncertain” were subsequently reviewed and discussed with a second reviewer until consensus was reached. This approach is consistent with narrative review methodology, where flexibility is required to accommodate heterogeneity in study designs and outcomes.

### 2.3. Inclusion and Exclusion Criteria

Studies were eligible if they:

- were published in peer-reviewed academic journals in English;
- included participants aged 3–18 years with a formal ASD diagnosis based on standardized tools such as the ADOS or ADI-R [33,34]; Although all included participants had a formal ASD diagnosis, several studies did not explicitly report the diagnostic instrument used. This reflects variability in reporting practices rather than absence of standardized diagnosis.
- examined FMSs, gross or fine motor skills, MC, or PMC as primary outcomes;
- employed quantitative, qualitative, or mixed-method designs, including cross-sectional, longitudinal, experimental, quasi-experimental, pilot, or review studies;
- and reported outcomes relevant to motor performance, perceived competence, physical activity participation, or developmental correlates.

Comparative studies including both ASD and typically developing (TD) children were included only when ASD data were reported separately.

Studies were excluded if they:

- focused on infants or adults outside the 3–18 age range;
- addressed domains unrelated to motor development (e.g., sensory processing, language, self-esteem);
- used interventions not directly targeting motor competence, such as aquatic therapy or horseback riding, whose primary aims relate to social or behavioral outcomes rather than structured motor skill acquisition [35,36];
- were not peer-reviewed (e.g., theses, conference abstracts);
- or lacked sufficient methodological detail or did not report motor outcomes.

Interventions such as aquatic therapy or horseback riding were excluded when their primary aims related to sensory regulation, social interaction, or behavioral outcomes rather than structured motor skill acquisition. This decision ensured conceptual coherence with the review’s focus on fundamental motor skills, motor competence, and perceived motor competence [37,38].

**Figure 1:**
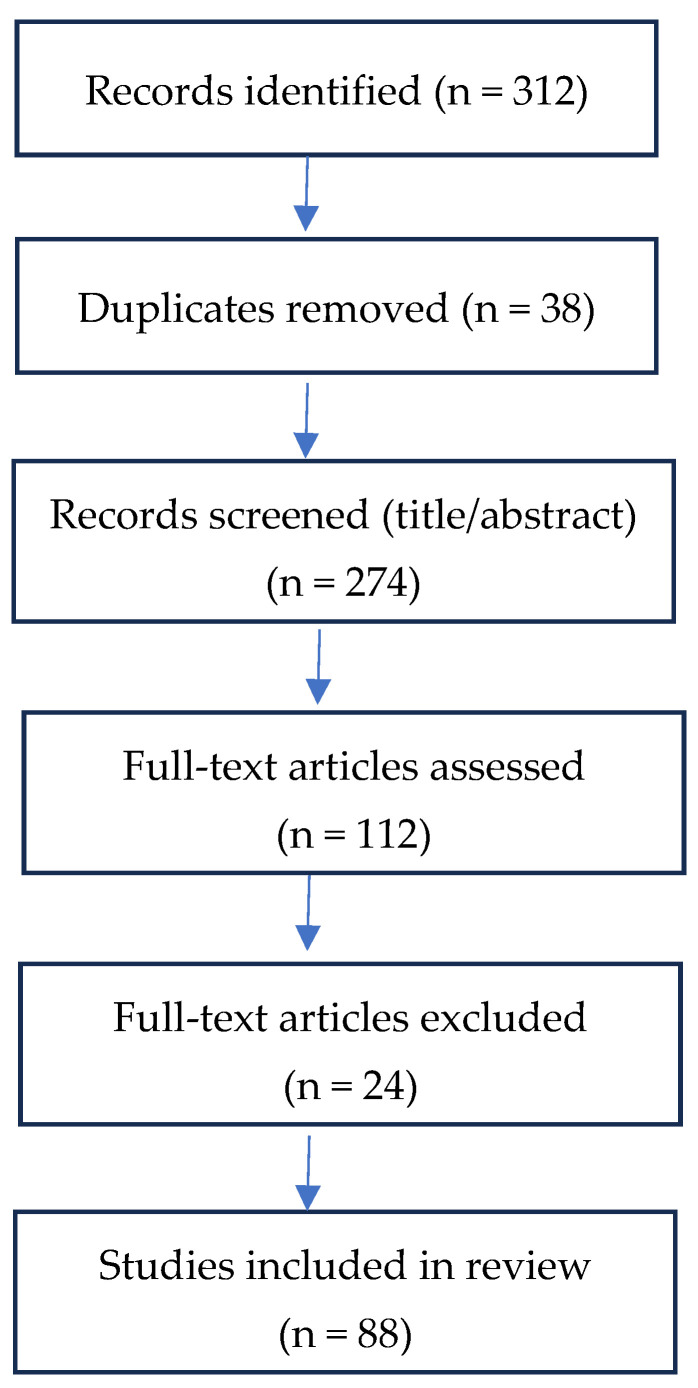
Study selection process, including inclusion and exclusion criteria.

The decision to exclude intervention modalities such as aquatic therapy or horseback riding was based on their primary therapeutic focus, which typically centers on sensory regulation, social interaction, or behavioral outcomes rather than structured motor skill acquisition. While these approaches may offer meaningful benefits for children with ASD, their aims and instructional methods differ substantially from interventions designed to target fundamental motor skills or motor competence. We acknowledge that this decision narrows the scope of the review and may limit the inclusion of studies reporting broader developmental outcomes; however, it ensures conceptual coherence with the review’s focus on FMSs, MC, and PMC. Figure 1 presents the study selection process, including inclusion and exclusion criteria.

Inclusion Criteria

- Peer-reviewed studies in English
- Participants aged 3–18 years
- Formal ASD diagnosis
- Outcomes related to FMSs, MC, or PMC
- Quantitative, qualitative, or mixed-method designs
- Reported motor-related outcomes

Exclusion Criteria

- Infants or adults outside 3–18 years
- No ASD diagnosis
- No motor-related outcomes
- Non–peer-reviewed sources
- Interventions not targeting motor competence
- Insufficient methodological detail

Clarifying Review Design

This manuscript is intentionally designed as a narrative review. Structured elements such as search terms, a flow diagram, and summary tables were included solely to enhance transparency and reader accessibility, rather than to emulate a systematic or scoping review framework. Accordingly, procedures typical of systematic reviews—such as independent dual screening, formal quality appraisal, or adherence to PRISMA-ScR—were not applied. This approach is consistent with narrative synthesis methodologies, which prioritize conceptual integration and interpretive depth over exhaustive procedural standardization.

Given this design, all interpretations—particularly those related to intervention effectiveness—should be viewed as tentative and context-dependent.

### 2.4. Data Extraction and Synthesis

Given the heterogeneity of study designs, assessment tools, and intervention approaches, a narrative synthesis was deemed the most appropriate method. This review is designed as a conceptually driven narrative synthesis informed by a structured search strategy. The purpose was not to conduct a systematic review, but to integrate diverse strands of evidence into a coherent developmental framework linking FMSs, MC, and PMC in children and adolescents with ASD [17,18]. For each included study, the following information was extracted: participant characteristics (age, sex, ASD severity), study design, assessment tools used (e.g., TGMD-2, MABC-2) [39,40], type and duration of intervention (if applicable), primary motor outcomes, and associations with PMC, physical activity, or developmental factors.

Studies were then organized into four thematic domains:

1. participant characteristics;
2. motor competence profiles;
3. intervention features and outcomes;
4. assessment tools.

This thematic structure allowed for integration across diverse methodologies while highlighting consistent patterns, discrepancies, and gaps in the literature.

A formal quality appraisal was not conducted, as the substantial heterogeneity across study designs, assessment tools, and intervention formats limits the applicability of standardized appraisal frameworks. Instead, methodological considerations were incorporated narratively throughout the synthesis, with attention to sample characteristics, measurement validity, and intervention structure. This approach aligns with the conceptual aims of the review and avoids imposing uniform criteria on studies with fundamentally different purposes and designs.

These methodological decisions inevitably shape the scope and interpretation of the review. By prioritizing studies that directly assess FMSs, MC, or PMC, the synthesis focuses on constructs central to motor development while acknowledging that broader therapeutic interventions may contribute to related outcomes. The narrative approach allows these methodological boundaries to be made explicit and supports a more transparent interpretation of the evidence.

The studies excluded during the screening process are provided in the Supplementary Material (Table S1), ensuring full transparency regarding study selection.

## 3. Results

The Results section aligns with the study aims, with subsections addressing Fundamental Motor Skills (FMSs), Motor Competence (MC), Perceived Motor Competence (PMC), intervention outcomes, and assessment tools. A total of 88 peer-reviewed studies met the inclusion criteria and were synthesized across four thematic domains: participant characteristics, motor competence profiles, intervention features and outcomes, and assessment tools. Although the studies varied widely in design and methodological rigor, several consistent patterns emerged.

### 3.1. Cross-Study Analytical Integration

Across the included intervention studies, several cross-study patterns became evident. Intervention dosage and duration varied substantially, ranging from brief 4-week protocols to multi-month programs. Higher-dosage interventions tended to report more consistent improvements in FMSs and MC, particularly when instructional methods emphasized structured, task-specific practice rather than general motor play. Studies employing individualized or small-group instruction also demonstrated clearer gains compared to those using large-group formats.

Differences in assessment tools contributed to variability in reported outcomes. Studies using standardized motor assessments (e.g., MABC-2, TGMD-2) generally identified more pronounced motor delays than those relying on researcher-developed checklists, which tended to yield more heterogeneous results. Variation in sample characteristics—including age range, ASD severity, and co-occurring conditions—further shaped performance patterns, with broader developmental profiles associated with greater variability.

Across both observational and intervention studies, recurring methodological patterns were identifiable. Interventions grounded in explicit motor instruction consistently produced measurable improvements, whereas less structured approaches yielded mixed or modest effects. Observational studies converged in documenting persistent FMSs and MC challenges in children with ASD, although the magnitude of these difficulties varied depending on assessment method and sample composition.

Taken together, these comparative observations provide a more integrated interpretation of the literature by highlighting both areas of consistency and sources of divergence across studies.

### 3.2. Participant Characteristics

Across the literature, samples were predominantly male, reflecting the higher prevalence of ASD in boys. Most studies focused on children between 5 and 13 years of age, with relatively few including adolescents—a gap that limits our understanding of motor development during later developmental stages. Reporting of ASD severity was inconsistent, and only a minority of studies provided detailed diagnostic information using standardized tools such as the ADOS or ADI-R [33,34]. In several studies, the diagnostic tool was not specified, even though participants were described as having a formal ASD diagnosis. This inconsistency reflects incomplete reporting rather than lack of standardized assessment. This variability makes it difficult to compare findings across studies or to examine how motor performance may differ by severity level or diagnostic subtype. Of the 88 included studies, 14 involved adolescents aged 13–18 years.

### 3.3. Motor Competence Profiles

Across the literature, children with ASD consistently demonstrate lower performance in both gross and fine motor skills compared with their typically developing peers. Common difficulties include reduced balance, limited coordination, and lower proficiency in locomotor and object-control tasks, patterns repeatedly documented across multiple studies [7,8,9,10,11]. Beyond these core motor challenges, several investigations have identified associations between motor competence and broader developmental outcomes, including social participation, adaptive functioning, and academic performance [9,13].

Although motor delays are evident throughout childhood, evidence concerning adolescents remains limited. The few studies that include older participants suggest that motor difficulties often persist into adolescence, yet developmental trajectories and age-related changes are not well understood [23]. Findings regarding associations between motor competence and factors such as physical activity or perceived competence are mixed, likely reflecting variability in measurement tools, sample characteristics, and study design [17,18].

Only five studies assessed Perceived Motor Competence (PMC), using instruments such as the PSPMSC, the Physical Self-Perception Profile, and the Pictorial Scale of Perceived Competence. Across these studies, children and adolescents with ASD reported lower perceived competence than their typically developing peers, and PMC was positively associated with actual motor competence. However, the strength of this relationship varied depending on age, assessment instrument, and methodological approach [13,18].

### 3.4. Intervention Features and Outcomes

Intervention studies varied widely in design, duration, and instructional approach. Most programs targeted FMSs through structured physical activity, adapted physical education, or task-specific training. Frequencies ranged from one to three sessions per week, with total durations spanning from five weeks to ten months. Interventions demonstrating the most consistent improvements included structured FMS programs, gymnastics-based training, and guided physical activity sessions. These approaches showed measurable gains in locomotor and object control skills, with some studies also reporting improvements in engagement and enjoyment [17,20,23,24].

Overall, interventions tended to report improvements in motor performance, particularly in locomotor and object control skills [17,20,23,24]. Gymnastics-based programs [31] and guided physical activity interventions [23,24] showed promising results for enhancing motor competence and engagement. A small number of studies also examined broader developmental outcomes. For example, a six-month adapted aerobic program demonstrated improvements not only in motor proficiency but also in BMI and cognitive abilities among students with ASD, underscoring the potential multidimensional benefits of movement-based interventions [41].

However, outcomes were not uniformly positive. Several studies reported modest or mixed effects, often influenced by small sample sizes, short intervention periods, or variability in instructional methods [23]. Few studies examined long-term retention of motor gains, and evidence for adolescents remains particularly sparse [20].

Across the intervention literature, several characteristics appeared to be associated with more positive outcomes. Programs that were structured, developmentally sequenced, and delivered with clear instructional routines tended to yield the most consistent improvements in locomotor and object control skills [20,23,24]. Interventions incorporating repetitive practice, visual supports, modeling, and task-specific feedback were particularly effective for children with ASD, who often benefit from predictable learning environments and explicit skill breakdown [17,18,21]. Gymnastics-based programs and guided physical activity sessions demonstrated notable gains in balance, coordination, and overall motor competence, while also enhancing engagement and enjoyment. Shorter interventions (5–8 weeks) produced measurable improvements, but longer programs (12 weeks or more) generally resulted in stronger and more stable effects [20,41]. These patterns offer practical guidance for educators and practitioners designing motor skill programs for children and adolescents with ASD.

### 3.5. Assessment Tools

Across the included literature, 11 different assessment instruments were used to evaluate fundamental motor skills, motor competence, or perceived motor competence, with the Test of Gross Motor Development-2 (TGMD-2) and Movement Assessment Battery for Children-2 (MABC-2) being the most frequently applied [39,40]. While these tools are widely used in motor development research, their psychometric suitability for ASD populations was not consistently addressed. Some studies highlighted challenges related to attention, comprehension, or behavioral regulation during testing, raising questions about validity and feasibility for certain subgroups.

Recent work has introduced alternative or adapted measures, including pictorial scales and observational checklists, to capture perceived competence or context-specific motor behaviors [13,17,27,32]. Although these tools offer promising avenues for more flexible assessment, they remain less standardized and require further validation.

Table 2 and Table 3 presents only the studies that specifically examined FMSs, MC, or PMC outcomes. Although 88 studies were included in the overall review, not all of them assessed these constructs directly; therefore, only the relevant subset of studies is displayed in the tables to enhance clarity and thematic coherence.

## 4. Discussion

The findings of this narrative review reinforce the growing recognition that motor difficulties are not peripheral or secondary features of autism spectrum disorder (ASD) but rather core developmental characteristics that influence children’s daily functioning in meaningful ways. Across the reviewed studies, children with ASD consistently demonstrated delays in both gross and fine motor skills, with particular challenges in locomotor and object control abilities [8,9,10,11]. These difficulties often persist across childhood and, based on the limited evidence available, may extend into adolescence as well. Although the developmental trajectories remain insufficiently understood, the consistency of these findings underscores the need to view motor development as a central component of ASD rather than an adjunct concern.

Given the narrative nature of the synthesis and the absence of a formal quality appraisal, the interpretation of findings has been revised to adopt a more cautious tone. Practical implications are now framed as tentative and context-dependent, reflecting the heterogeneity of study designs, intervention formats, and assessment tools.

To strengthen the conceptual grounding of the review, the proposed integrative developmental model has been further elaborated. The model conceptualizes motor performance as emerging from dynamic interactions among cognitive processes (e.g., planning, attention), motivational factors (e.g., perceived competence, engagement), and environmental opportunities (e.g., structured practice, instructional support). These components are mutually reinforcing: cognitive resources shape motor learning, motivational states influence persistence and participation, and environmental affordances determine the quality and frequency of motor experiences.

This framework was used explicitly to guide the interpretation of findings. For example, consistent improvements in structured, task-specific interventions align with the model’s emphasis on environmental scaffolding, while variability in PMC outcomes reflects the interplay between motivational beliefs and motor performance. Similarly, differences in assessment outcomes can be understood through the cognitive demands embedded in each tool.

**Figure 2:**
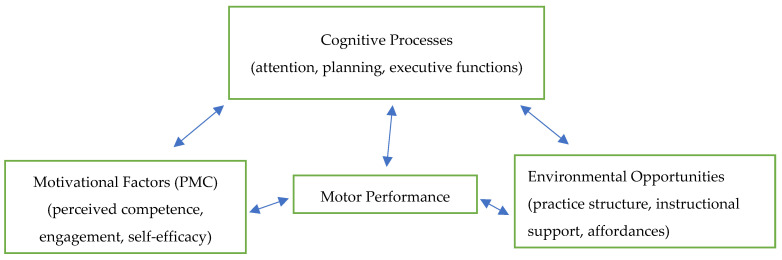
Integrative developmental model illustrating dynamic, bidirectional relationships among motor performance, cognitive processes, motivational factors, and environmental opportunities. Bidirectional arrows represent reciprocal influences across domains.

To enhance conceptual clarity, we developed a visual representation of the proposed integrative developmental model (Figure 2). The diagram illustrates the dynamic, bidirectional relationships among motor performance, cognitive processes, motivational factors, and environmental opportunities. This visual framework supports a more coherent interpretation of the heterogeneous findings and clarifies how these domains interact to shape motor outcomes in children with ASD.

Because the review did not include a formal quality appraisal, intervention findings are interpreted cautiously, and their generalizability should be considered limited. Heterogeneous study designs, small pilot samples, and variability in assessment tools further reinforce the need for tentative, context-dependent conclusions.

All studies included in the summary tables were re-checked for alignment with the stated inclusion criteria, and minor inconsistencies between tables and narrative descriptions were corrected to ensure internal coherence.

A key contribution of this review is the integration of fundamental motor skills (FMSs), motor competence (MC), and perceived motor competence (PMC) within a unified developmental framework. While these constructs are frequently examined in isolation, the evidence suggests that they are deeply interconnected. FMSs provide the building blocks for more complex movement patterns; MC reflects the quality, coordination, and adaptability of these skills; and PMC shapes children’s motivation, confidence, and willingness to participate in physical activity [13,18,27,32]. For children with ASD, difficulties in any of these domains may create a reinforcing cycle: reduced motor proficiency limits participation, which in turn restricts practice opportunities and further constrains motor development. This cycle may be intensified by cognitive factors, as emerging neuropsychological evidence highlights the role of visuospatial working memory, attention, planning, and decision-making in motor performance [15].

Intervention studies provide encouraging, though not unequivocal, evidence. Structured, repetitive, and task-specific programs tend to yield improvements in locomotor and object-control skills [17,20,23,24]. Gymnastics-based interventions [31] and guided physical activity programs [23,24] appear particularly promising, enhancing both motor competence and engagement. Complementary approaches such as Tai Chi have also demonstrated improvements in balance and coordination among children with ASD [42]. Evidence from other neurodevelopmental populations further supports the value of structured movement programs; for example, a Greek traditional dance intervention improved both motor proficiency and social skills in children with developmental coordination disorder [43]. Meta-analytic findings further support the effectiveness of structured exercise interventions for improving FMSs in children with ASD and/or ADHD [44]. Importantly, some interventions report broader developmental benefits, including gains in BMI, executive functioning, social behavior, and overall quality of life [16,28,29].

Despite these positive trends, several limitations temper the strength of the evidence. Many studies relied on small samples, short intervention durations, or non-randomized designs, making it difficult to draw firm conclusions about effectiveness or generalizability. Long-term follow-up data are scarce, leaving open questions about the sustainability of motor gains. Adolescents remain particularly underrepresented, despite the fact that motor challenges may become more salient during this developmental period as social expectations and physical demands increase [23]. Across the included studies, methodological rigor varied considerably. Common limitations included small sample sizes, inconsistent reporting of diagnostic tools [33,34], heterogeneity in assessment instruments [39,40], short intervention durations, and challenges related to attention or behavioral regulation during testing. These factors constrain comparability across studies and should be considered when interpreting the findings.

Adolescence represents a distinct developmental stage that warrants explicit consideration when interpreting motor competence outcomes in individuals with ASD. Although much of the literature focuses on younger children, available evidence indicates that motor difficulties frequently persist into later developmental stages and may even intensify as social, cognitive, and environmental demands increase [8,9,10,11]. During adolescence, expectations for autonomous participation in physical activity, organized sports, and peer-based movement contexts become more pronounced, yet autistic adolescents often report challenges that limit their engagement and confidence in school-based physical education [45]. These limitations are closely associated with reduced physical activity participation, lower perceived motor competence, and increased social self-consciousness, factors that may further restrict opportunities for practice and skill refinement [27]. Importantly, adolescent-specific interventions remain scarce, highlighting the need for research that addresses the unique developmental, psychosocial, and motivational characteristics of this age group rather than extrapolating findings from younger children. The limited representation of adolescents in the reviewed literature restricts our understanding of developmental trajectories during this period. As physical, social, and cognitive demands increase, motor challenges may become more pronounced, underscoring the need for age-specific research and tailored intervention approaches [23].

Methodological inconsistencies also complicate interpretation. The TGMD-2 and MABC-2 remain the most commonly used assessment tools, yet their suitability for ASD populations is not consistently addressed [39,40]. Challenges related to attention, comprehension, and behavioral regulation during testing raise concerns about the validity of standardized assessments for certain subgroups. Emerging tools—such as pictorial scales and context-specific observational measures—offer promising alternatives but require further validation [27,32]. Recent comparative work further underscores the need for sensitive assessment tools, as children with ASD consistently score lower than their typically developing peers across all FMS domains [8,9,10,11]. Moreover, studies linking MC with nutrition, BMI, and social functioning highlight the multidimensional nature of motor competence [28,29].

Taken together, the findings highlight the need for more rigorous, developmentally sensitive research that examines motor competence across childhood and adolescence, integrates both actual and perceived competence, and considers the broader educational and social contexts in which motor skills are learned and practiced. Such work is essential for informing inclusive physical education and adapted physical activity settings, where educators must respond to diverse motor, sensory, and behavioral profiles. The evidence reviewed here underscores that motor development is not merely a physical domain but a multidimensional construct with implications for cognitive, social, and emotional well-being.

### Implications for Practice

The findings of this review carry important implications for professionals working in inclusive physical education and adapted physical activity settings. Motor difficulties in children and adolescents with ASD are not peripheral concerns but core developmental features that shape participation, confidence, and social engagement. Early identification of motor delays is therefore essential. The use of validated assessment tools such as the TGMD-2 and MABC-2 can support accurate profiling of strengths and needs, although practitioners must remain mindful of the attentional, behavioral, and comprehension demands these instruments may place on learners [39,40].

Instructional approaches should prioritize individualized and developmentally sensitive teaching. Evidence consistently shows that structured, repetitive, and task-specific practice enhances motor performance [17,20,23,24]. Creating learning environments with clear routines, predictable task sequences, and frequent opportunities for practice can help students with ASD engage more confidently. Visual supports, simplified task breakdowns, and explicit demonstrations are particularly valuable for learners who experience challenges with attention, planning, or working memory [2,3,15].

Given the strong links between perceived motor competence, motivation, and participation, practitioners should also aim to cultivate positive movement experiences. Activities that allow for success, autonomy, and gradual skill progression can strengthen students’ confidence and willingness to participate. Peer-supported tasks and cooperative activities may further promote social interaction while reducing the performance pressure often associated with competitive settings.

The broader developmental benefits of physical activity should not be overlooked. Well-designed movement programs have the potential to support not only motor proficiency but also executive functioning, social skills, and overall quality of life [16,18,29]. Evidence from gymnastics-based and structured exercise interventions reinforces their value for children with ASD [17,31], while complementary approaches such as Tai Chi have also demonstrated improvements in balance and coordination [42]. Collaboration among physical educators, occupational therapists, psychologists, and families can enhance intervention consistency and effectiveness.

Finally, the limited evidence available for adolescents underscores the need for age-appropriate programming. As social expectations and physical demands increase during adolescence, motor difficulties may become more salient and may influence participation in school and community activities. Tailored interventions that address both skill development and psychosocial factors—such as self-esteem, peer relationships, and perceived barriers to participation—are essential for supporting continued engagement in physical activity during this critical developmental period [45].

## 5. Conclusions

Given the methodological variability and the narrative nature of the synthesis, the conclusions of this review should be interpreted with caution. Although many intervention studies report short-term improvements, the wide variation in methods, assessment tools, and sample characteristics makes it difficult to draw firm conclusions. Evidence involving adolescents is still limited, leaving important questions about how motor skills develop during later stages of childhood.

Future research would benefit from more longitudinal designs, clearer and more consistent assessment practices, and interventions that take into account both motor performance and motivational factors. Strengthening these areas can help educators and practitioners design programs that better support participation, confidence, and motor development in children and adolescents on the autism spectrum.

## Figures and Tables

**Table 1:** Full Search Strings Used in Each Database.

Database	Full Search String	Notes
**PubMed**	(“autism spectrum disorder” OR ASD OR autism) AND (“fundamental motor skills” OR “motor competence” OR “gross motor skills” OR “fine motor skills” OR “perceived motor competence”) AND (child OR children OR adolescent OR youth)	March 2020–October 2025; English; peer-reviewed
**ScienceDirect**	TITLE-ABSTR-KEY (“autism spectrum disorder” OR ASD) AND (“fundamental motor skills” OR “motor competence” OR “motor skills” OR “perceived motor competence”) AND (child* OR adolescen*)	March 2020–October 2025
**ProQuest**	(autism OR “autism spectrum disorder” OR ASD) AND (“motor competence” OR “fundamental motor skills” OR “gross motor skills” OR “fine motor skills” OR “perceived motor competence”) AND (child* OR adolescen*)	Peer-reviewed filter applied
**Google Scholar**	“autism spectrum disorder” “motor competence” “fundamental motor skills” “perceived motor competence”	First 200 results screened; duplicates removed

**Table 2:** Fundamental Motor Skills (FMSs): Study Characteristics and Key Findings.

Study	Participants (N, Age, Gender)	Diagnosis	Research Design	Intervention	Measurement Tool	Key Findings/Outcomes
Bremer et al. (2014) [20]	8 children, 4 years old, 7 boys/1 girl	ASD	Quasi -experimental, wait-list control	Group 1 1/week for 60 min/for 12 weeks Group 2 2/week for 60 min/for 6 weeks	PDMS-2, MABC	Improvements in locomotor and object control skills; higher gains in higher-frequency group
Bremer and Lloyd (2016) [21]	5 children, 3–7 years, 4 boys/1 girl	4 ASD, 1 ASD-like behaviors	Multiple methods	3/week for 45 min/for 12 weeks	TGMD	Gains in FMS performance; strong engagement and responsiveness to structured instruction
Pan et al. (2017) [22]	33 children, 6–12 years, mixed gender	ASD	Experimental (pre–post)	Physical activity program, 2–3 times per week for 12 weeks	Motor assessments and cognitive tests	Improvements in motor performance and executive functioning; increased engagement in physical activity
Ketcheson et al. (2017) [23]	20 children, 4–6 years, 15 boys/5 girls	ASD	Quasi-experimental	5/week for 4 h/for 8 weeks	TGMD	Significant improvements in FMS proficiency; strong gains in object control
Dong et al. (2024) [24]	60 children, 7–10 years, balanced gender	ASD & TD	Cross-sectional	No intervention (comparison study)	TGMD-2	ASD group scored significantly lower than TD peers in all FMS domains

**Table 3:** Motor Competence (MC) and Perceived Motor Competence (PMC): Study Characteristics and Key Findings.

Study	Participants (N, Gender, Age)	Diagnosis	Research Design	Intervention	Measurement Tool	Key Findings/Outcomes
Barnett et al. (2011) [25]	215 adolescents, 16.4 ± 0.6 yrs, 48% boys/52% girls	Typical Development (TD)	Cross-sectional	Responses based on a typical week of PA (3 days-training)	Get Skilled, Get Active (MC) & Physical Self-Perception Profile (PMC)	Strong positive association between MC and PMC
Castelli &Valley, (2007) [26]	230 children, 7–12 yrs, 140 boys/90 girls	TD	Multiple methods	7-day recall, 45 min during physical activity program	South Carolina Physical Education Assessment Program (SCPEAP)	Higher MC linked to higher PA levels
Clark et al. (2018) [27]	58 children, 9.5 yrs, 29 boys/29 girls	TD	Cross-sectional	10 min PSPMSC& 20 min TGMD-2	The Pictorial Scale of Perceived Movement Skill Competence for Young Children-PSPMSC (PMC) & TGMD-2 (MC)	PMC positively correlated with MC
Liu & Breslin (2013) [10]	51 children, 7–12 yrs, 46 boys/5 girls	ASD	Multiple Methods	Extensive training prior administration	MABC-2	Lower MC scores in ASD; attention influenced performance
Liu et al. (2014) [11]	51 children, 7–12 yrs, 46 boys/5 girls	ASD & TD	Cross-sectional	TGMD-2 motor performance comparison	TGMD-2	ASD group significantly lower in MC
Liu et al. (2019a) [28]	N/A (age not specified)	ASD	Cross-sectional	Correlation of nutrition and BMI with MC	Medicina motor competence scale	MC associated with BMI and nutrition
Liu et al. (2019b) [29]	N/A (age not specified)	ASD	Cross-sectional	Correlation of MC with social functioning	J Phys Educ Sport metrics	MC associated with social functioning
Peers et al. (2020) [30]	860 children, 10.9 yrs, 47.7% girls	TD	Cross-sectional	10 min PSPMSC & 20 min TGMD-2	The Pictorial Scale of Perceived Movement Skill Competence-PSPMSC (PMC) & TGMD-2 (MC)	Strong MC–PMC relationship across large sample
Quito et al. (2025) [31]	20 boys, 6–9 yrs	ASD	Pilot experimental	Gymnastics-based training, 2/week for 8 weeks	Movement ABC & observational checklist	Improvements in MC and engagement
Robinson (2010) [32]	119 children, 4 yrs, 65 boys/54 girls	TD	Cross-sectional	10 min PSPC & 20 min TGMD-2	The Pictorial Scale of Perceived Competence & Social Acceptance-PSPC (PMC) & TGMD-2 (MC)	PMC linked to early MC development

## Data Availability

The data presented in this study are available upon request from the corresponding author.

## Supplementary Materials

The following supporting information can be downloaded at: https://www.mdpi.com/article/10.3390/children13040520/s1, Table S1: Studies excluded from the final synthesis. References [46,47,48,49,50,51,52,53] are cited in the supplementary materials.
