# Supplementary material for: Fundamental Motor Skills and Motor Competence in Children and Adolescents with Autism Spectrum Disorder (ASD): A Narrative Review
Source: Children (Basel). 2026 Apr 8;13(4):520. doi: 10.3390/children13040520 (PMC13114944; doi:10.3390/children13040520)
Supplement: Supplementary file 1 [file children-13-00520-s001.zip › children-4197151-supplementary.pdf]

Table S1. Studies excluded from the final synthesis

Study	Title	Participants (N, age, gender)	Diagnosis	Measurement/ Tools	Reason for Exclusion
**Lindsay et al., (2006)** [46]	Dietary status and impact of risperidone on nutritional balance in children with autism: A pilot study	20 children, 19 boys and 1 girl	ASD	Quantitative Food Frequency Questionnaire (FFQ)	No motor outcomes assessed
**Tse, (2019)** [47]	Effects of attentional focus on motor learning in children with autism spectrum disorder	65 children, 48 boys and 17 girls	ASD, Asperger	Experimental, TGMD-2, Throwing performance	Focus on attentional focus, not FMS/MC/PMC
**Sarabzadeh, Bordbar Azari & Helalizadeh, (2019) [42]**	The effect of six weeks of Tai Chi Chuan training on the motor skills of children with autism spectrum disorder	18 children, 14 boys & 4 girls	ASD	Tai Chi Chuan	Intervention not targeting structured motor skill acquisition
**Bedford, Pickles& Lord, (2016)** [48]	Early Gross Motor Skills Predict the Subsequent Development of Language in Children with Autism Spectrum Disorder	209 participants, 170 boys, 39 girls	ASD	Vineland adaptive behavior scales (VABS-II), Mullen scales of early learning (MSEL), Autism diagnostic interview-revised	Predictive developmental study; no FMS/MC/PMC outcomes
**Pan, (2011)** [49]	The efficacy of an aquatic program on physical fitness and aquatic skills in children with and without autism spectrum disorders	30 participants, 15ASD & 15 TD	ASD & Typically Developed siblings	14 weeks aquatic program	Focus on aquatic skills, not motor competence
**Leonard, Elsabbagh, Hill, & Basis Team, (2014)** [50]	Early and persistent motor difficulties in infants at-risk of developing autism spectrum disorder: A prospective study	44 at-risk infants & 50 low-risk infants	ASD	Autism Diagnostic Observation Schedule – Generic & Autism Diagnostic Interview – Revised	Infants (<3 years), outside age range
**Gabriels, Pan, Dechant, Agnew, Brim & Mesibov, (2015)** [51]	Randomized Controlled Trial of Therapeutic Horseback Riding in Children and Adolescents with Autism Spectrum Disorder	85 participants	ASD	Therapeutic Horseback riding	Primary outcomes social/behavioral, not motor competence
**Hilton et al., (2014)** [52]	Effects of Exergaming on Executive Function and Motor Skills in Children with Autism Spectrum Disorder: A Pilot Study	-	ASD	Makoto arena training intervention	Executive function focus; insufficient motor outcome detail

**Alaniz, Rosenberg, Beard, & Rosario, (2017)** [53]	The Effectiveness of Aquatic Group Therapy for Improving Water Safety and Social Interactions in Children with Autism Spectrum Disorder: A Pilot Program	7	ASD	Aquatic therapy program on water safety and social skills	Focus on water safety/social skills, not FMS/MC/PMC
